# Transforming Growth Factor-*β* and Oxidative Stress in Cancer: A Crosstalk in Driving Tumor Transformation

**DOI:** 10.3390/cancers13123093

**Published:** 2021-06-21

**Authors:** Valeria Ramundo, Giuliana Giribaldi, Elisabetta Aldieri

**Affiliations:** Department of Oncology, University of Torino, 10126 Torino, Italy; valeria.ramundo@unito.it (V.R.); giuliana.giribaldi@unito.it (G.G.)

**Keywords:** TGF-*β*, oxidative stress, EMT

## Abstract

**Simple Summary:**

Metabolic changes in tumor microenvironment play a critical role in cancer, related to the accumulated alterations in signaling pathways that control cellular metabolism. Cancer metabolic deregulation is related to specific events such as the control of oxidative stress, and in particular the redox imbalance with aberrant oxidant production and/or a deregulation of the efficacy of the antioxidant systems. In cancer cells, different cytokines are involved in the development and/or progression of cancer; among these cytokines, the transforming growth factor *β* (TGF-*β*) is central to tumorigenesis and cancer progression. In tumor cells, it has been demonstrated that there is a close correlation between oxidative stress and TGF-*β*; this crosstalk strongly contributes to tumorigenesis, both in tumor development and in mediating its invasiveness. This review is addressed to better understanding this crosstalk between TGF-*β* and oxidative stress in cancer cell metabolism, in an attempt to improve the pharmacological and therapeutic approach against cancer.

**Abstract:**

Cancer metabolism involves different changes at a cellular level, and altered metabolic pathways have been demonstrated to be heavily involved in tumorigenesis and invasiveness. A crucial role for oxidative stress in cancer initiation and progression has been demonstrated; redox imbalance, due to aberrant reactive oxygen species (ROS) production or deregulated efficacy of antioxidant systems (superoxide dismutase, catalase, GSH), contributes to tumor initiation and progression of several types of cancer. ROS may modulate cancer cell metabolism by acting as secondary messengers in the signaling pathways (NF-kB, HIF-1*α*) involved in cellular proliferation and metastasis. It is known that ROS mediate many of the effects of transforming growth factor *β* (TGF-*β*), a key cytokine central in tumorigenesis and cancer progression, which in turn can modulate ROS production and the related antioxidant system activity. Thus, ROS synergize with TGF-*β* in cancer cell metabolism by increasing the redox imbalance in cancer cells and by inducing the epithelial mesenchymal transition (EMT), a crucial event associated with tumor invasiveness and metastases. Taken as a whole, this review is addressed to better understanding this crosstalk between TGF-*β* and oxidative stress in cancer cell metabolism, in the attempt to improve the pharmacological and therapeutic approach against cancer.

## 1. Introduction

Cancer metabolism involves different changes at cellular level, and altered metabolic pathways have been demonstrated to be involved in tumorigenesis and invasiveness.

Metabolic changes in the tumor microenvironment play a critical role in cancer, especially in the uncontrolled cell proliferation related to accumulated alterations in the signaling pathways that control cellular metabolism, which in turn sustain enhanced cell proliferation. Thus, to achieve and sustain that proliferative capacity, cancer cells must induce or modulate some metabolic pathways [1].

Cancer metabolic deregulation is related to specific events such as the control of oxidative stress, in particular the redox imbalance with aberrant oxidant production and/or a lower or higher efficacy of antioxidant systems, and the epithelial to mesenchymal transition (EMT) [2,3], a physiological event widely involved in tumor development and metastases. Both of these events are differently linked to the cytokine transforming growth factor *β* (TGF-*β*), whose role in tumorigenesis has been extensively studied [4], although it has not yet been fully clarified; there is a close correlation between TGF-*β* and oxidative stress, a strong crosstalk that can contribute to the development and/or progression of the tumor.

The purpose of this review is to focus on the role of TGF-*β* as a key molecule in cancer and its interplay with oxidative stress produced at a cellular level, considering that both are part of the complex cascade of events involved in cancer cellular metabolism.

## 2. Transforming Growth Factor *β* (TGF-*β*) in Cancer Metabolism

In cancer cells, different cytokines are involved in the development and/or progression of cancer. Among these cytokines, TGF-*β* is central to tumorigenesis and cancer progression, although depending on the cancer stage [4]. 

TGF-*β* belongs to a large family of more than 40 proteins, which are organized in several subfamilies. Among the different TGF-*β* isoforms, at least three genetically distinct ones are particularly expressed, called TGF-*β*1, TGF-*β*2, and TGF-*β*3, with a high homology, but collectively referred to as TGF-*β*. TGF-*β* binds to its receptor on the plasma membrane and activates downstream signaling by phosphorylation of different mediators, mainly SMAD proteins, which are the main effector molecules in the TGF-*β* signaling pathway [5]. The interaction between SMAD and other downstream proteins in turn controls the expression of specific target genes and the regulation of nuclear or cytoplasmic proteins. An alternative pathway, not mediated by SMAD, is also known [6], and it has been demonstrated to involve in particular the GTPases Ras and Rho and the mitogen-activated protein kinases (MAPK), all of which have been widely shown to be activated by TGF-*β* [7].

TGF-*β* has been demonstrated to have a dual role in tumor progression: on the one hand, TGF-*β* acts as a tumor suppressor, especially in the early stages of tumor development, where TGF-*β* promotes apoptosis and inhibits the proliferation of cancer cells [4]. On the other hand, in the advanced stage of the tumor, TGF-*β* has a pro-tumorigenic effect, promoting genomic mutations and events associated with the malignant progression of cancer, such as EMT, angiogenesis, inhibition of the immune response, cell motility, and thus the promotion of metastases [4,8]. At a DNA level, many genetic and epigenetic alterations have been associated in different types of cancer with variations in the TGF-*β* signaling, both in suppression and tumor promotion. At a clinical level, in particular, TGF-*β* expression is significantly increased during tumor progression, and this event often is correlated to a poor prognosis [4].

Furthermore, it seems that during tumor progression TGF-*β* acts differently, not only in correlation to the stage in carcinogenesis, but also according to the different reactivity of the tumor cells; this response may be associated with various factors, independently or related to TGF-*β* and its receptor expression, the availability of downstream signaling components, evasion of the immune response, stimulation of inflammation, and recruitment of cells that promote tumor growth [4]. Above all, some studies have shown that TGF-*β* expression is often increased in cell lines and tumor tissues compared to normal cells or tissues, while other studies have shown that the growth inhibition induced by TGF-*β* in non-transformed cells is often impaired in carcinomas [9], and in response to TGF-*β*, normal cells increase migration and invasiveness, especially via EMT, thus promoting cancer progression [10].

Therefore, several tumors express high levels of TGF-*β,* and this event correlates with a greater aggressiveness of the tumor and consequently a poor clinical prognosis [9]. Some alterations of key components involved in TGF-*β* signaling have been identified in association with oncogenic mutations, among which are those affecting SMAD, particularly the SMAD4 gene [11], which has been shown to be the most frequent alteration playing a key role in carcinogenesis, as in pancreatic ductal adenocarcinoma and colorectal and gastrointestinal cancers [12,13,14,15]. Finally, TGF-*β* acts as a potent immunosuppressive cytokine in cancer cells, enabling these cells to escape the surveillance exerted by the immune system, thereby promoting tumor growth and metastasis [16].

## 3. Oxidative Stress in Cancer Metabolism

Reactive oxygen species (ROS) are small molecules derived from oxygen and constantly generated by the body, such as superoxide anion, hydrogen peroxide, and hydroxyl radicals [17]. At a cellular level, ROS are constantly generated by enzymatic complexes or by reduction-oxidation (redox) reactions, which are particularly important, in the latter case, for keeping a correct redox balance in biological systems [18], especially at the level of the mitochondria [19]. Therefore, mitochondrial oxygen metabolism is linked to ROS generation [20], which, however, at high levels can damage cells by oxidizing nucleotides, proteins, and lipids, thus compromising cell survival. This failure in the maintenance of a correct redox homeostasis is commonly named “oxidative stress”.

Therefore, in physiological conditions the ROS produced are constantly eliminated by ROS scavenging systems, thus maintaining redox homeostasis. At a cellular level, this physiological redox homeostasis is maintained by different enzymes and molecules involved in the antioxidant defense, such as superoxide dismutase (SOD), catalase (CAT), and the reduced glutathione (GSH), a small peptide which controls cellular detoxification; ROS produced by the cellular metabolism are especially eliminated by these scavenging systems.

In cancer cells, the control of oxidative stress is strongly deregulated, on the one hand leading to an excessive ROS production, which contributes to the oxidative damage of proteins, lipids, and particularly DNA [21], and so promoting cellular damage and mutations that could all contribute to tumorigenesis; and on the other hand by hyperactivating the antioxidant defense systems, thus making cancer cells more resistant to counteracting oxidative stress, which is also associated with chemotherapy [22].

### 3.1. Redox Homeostasis in Cancer Metabolism

Cancer cells exhibit aberrant redox homeostasis. This redox imbalance, due to aberrant ROS production and/or antioxidant deregulated functionality, contributes to tumor progression and is a hallmark of several types of cancer [21,23]. ROS accumulation, derived from excessive oxidative stress, deregulates the antioxidant defense system, not only in cancer cells, but also when associated with various diseases, such as fibrosis [24].

Several metabolic effects have been related to tumor-associated redox imbalance: increased basal metabolic activity, mitochondrial and peroxisomal dysfunctions, deregulated cellular receptors, oncogenes upregulation, and cyclooxygenase, lipoxygenase and thymidine phosphorylase activation. These effects are in turn diversified according to the state of the tumor cells and their crosstalk with stroma and immune cells, as well as being associated with a higher activity of the antioxidant cellular system [21].

ROS have a dual role in cancer: high levels of ROS are cytotoxic but they are also pro-tumorigenic [25], as the hyperproliferation of cancer cells is accompanied by a high production of ROS; however, in this way, cancer cells are able to adapt themselves to thrive in conditions that can counteract this oxidative stress, by deregulating the cellular antioxidant defense systems and, at the same time, avoiding the ROS thresholds that would trigger apoptosis [26,27].

Concerning the antioxidant functionality in cancer cells, some of the antioxidant systems involved in detoxification from ROS are upregulated, thus helping to increase the resistance of cancer cells against oxidative stress, making the cancer cells also resistant to the toxicity exerted by chemotherapy [21]. Furthermore, ROS can act both at the level of early tumor development and metastases, thus working as secondary messengers in the activation and maintenance of signaling pathways involved in cell proliferation, particularly through NF-kB or HIF-1*α*, survival, angiogenesis, and EMT [28].

In many tumors, a strong redox imbalance is correlated to an increased cellular metabolic activity, particularly when associated to the NADPH oxidase (NOX) protein family [29].

#### NOX in Cancer Metabolism

The NOX protein family is one of the main sources of ROS. It is composed of five enzymes, identified as NOX 1–5, which are similar in their structure and in their catalytic function, and all mediating the reduction of molecular oxygen using NADPH as an electron donor [29]. ROS derived from NOX can act by modulating various metabolic events, in particular gene expression, proliferation, cell migration, and angiogenesis [30]. In cancer cells, it has been demonstrated that oxidative stress leading to tumor initiation or progression may result from overproduction of ROS by members of NOX [31]. Among the different NOX isoforms, NOX 2 and 4 when deregulated have been associated with cancer; particularly, NOX4 overexpression has been shown in different types of cancers, such as renal and pancreatic cancers, and in glioblastoma and invasive breast cancers [31]. However, conflicting evidence also suggests a role for NOX4 as a tumor suppressor: it has been shown that genetic deletion of NOX4 enhances cancerogen-induced formation of solid tumors [32]; moreover, NOX4 has been demonstrated to inhibit hepatocyte proliferation and liver cancer progression [33].

### 3.2. Antioxidant Systems in Cancer Metabolism

The maintenance of redox homeostasis between the generation and neutralization of ROS is guaranteed by the antioxidant defense systems, in order to protect the macromolecules from oxidative damage. The main antioxidant defense systems are both enzymes, superoxide dismutase (SOD) and catalase (CAT), and molecules such as reduced glutathione (GSH) in turn is kept active in its reduced form by enzymes such as glutathione peroxidase, glutathione reductase, and glutathione-S-transferase [19,20]. The expression levels of these antioxidant enzymes and molecules, as well as the presence of genetic polymorphisms, have frequently been associated with increased risk of cancer. Therefore, to counteract oxidative stress, cancer cells will deregulate these antioxidant systems, as described in the subsequent chapters.

#### 3.2.1. Superoxide Dismutase (SOD) in Cancer Metabolism

Among the main enzymes involved in antioxidant cellular defense, SOD, which is dependent on metal ions, is essential. SOD protects cells from oxidizing toxic products produced during aerobic respiration; this enzyme is involved in the conversion of superoxide radicals into oxygen and it has been shown that some defects in SOD activity are related to various cancers [34], although these data are often contradictory with each other; reduced SOD activity has been observed in brain tumor patients compared to normal subjects, while high SOD levels have been demonstrated to be associated with other cancers, such as breast and laryngeal carcinomas [35]. In general, reduced SOD activity is often associated with an increase in intracellular H2O2 levels, which would first favor DNA damage and consequently cancer development [36]. In tumors, a decreased SOD activity has been observed in the tumor tissue of patients with bladder cancer compared to the enzymatic activity present in benign tumors [37]. Furthermore, SOD expression has been shown to be significantly lower in invasive carcinomas than in superficial ones [38].

#### 3.2.2. Catalase (CAT) in Cancer Metabolism

CAT is an ubiquitous enzyme that physiologically catalyzes the decomposition of H2O2 to water and oxygen: CAT protects cells from excessive ROS production, thus preventing H2O2 accumulation. In many tumors, a decreased CAT expression and activity have been reported [34]: an altered CAT expression has been widely demonstrated in association with different types of cancers compared to their normal counterparts, as reported by some authors, who have shown an increased expression of CAT in tumors [39]. The regulation of CAT expression in cancer cells is a complex process, where various mechanisms appear to be involved: a recent review [40] explored these different crucial mechanisms in the CAT regulation, mainly via Akt/PKB in the PI3K signaling pathway in cancer cells.

#### 3.2.3. Reduced Glutathione (GSH) in Cancer Metabolism

GSH is the most important non-enzymatic antioxidant in cells, and it has been found to be essential in several cellular antioxidant systems. In normal cells, glutathione oxidation-reduction is the major intracellular pathway involved in ROS detoxification. For this reason, it is coupled to NADPH reduction-oxidation, thus maintaining GSH in its active reduced form and driving ROS detoxification [41]. A decreased GSH level is associated with a decreased defense against oxidative stress at a cellular level, while high levels of GSH increase the cellular antioxidant capacity and resistance to oxidative stress, with the latter condition particularly evident in many cancers. To date, high basal levels of GSH have been observed in many cancers, such as breast, ovarian, lung, and head and neck cancers [42]. Therefore, GSH plays an important role in tumors: many papers showed that increased GSH cellular levels are required for both initiation and tumor proliferation and invasiveness [43], while its implications in cancer have been used for improving chemotherapy [44].

### 3.3. ROS as Secondary Messengers in Cancer Metabolism

At a cancer level, ROS have been shown to be involved in both tumor development and progression, depending on whether these radicals act at the DNA level, promoting mutations, or in the regulation of intracellular signaling pathways involved in proliferation and survival [22]. ROS may modulate cancer cell metabolism by acting as secondary messengers in the signaling pathways involved in cellular proliferation and metastasis, such as those mediated by nuclear factor kB (NF-kB) and hypoxia inducible factor 1 *α* (HIF-1*α*).

#### 3.3.1. ROS and NF-kB

NF-kB is a transcription factor involved in the regulation of cellular proliferation, growth, and apoptosis, as well as in the modulation of inflammatory and pathological events, including in particular cancers [45]. The constitutive activation of NF-κB has been frequently observed in many tumors, and the upregulation of the NF-κB transcription pathway has been shown to promote most of the underlying characteristics of cancer [46]. NF-kB, in addition to its active role in tumor progression and metastasis, also regulates the transcription of genes that suppress apoptosis in tumor cells and increase inflammation in the tumor microenvironment, which is why NF-κB has recently been characterized as a key factor in tumor cell metabolism [46].

It is well known that ROS modulates NF-κB response and its target genes, by attenuating ROS to promote survival. One of the most important ways in which NF-κB activity influences ROS levels is via increased expression of antioxidant proteins [47]: SOD is the main antioxidant enzyme controlled by NF-κB, and glutathione S-transferase and glutathione peroxidase-1 are also upregulated by oxidative stress through NF-κB, as is heme oxygenase [47]. Since NF-κB is important in inflammation, some enzymes that promote the production of ROS are also regulated as its targets, especially in cells of the immune system. In particular, during the inflammatory process, expression of the phagocytic NOX2 is induced by NF-κB. In general, ROS stimulates the NF-κB pathway in the cytoplasm, but also inhibits NF-κB activity in the nucleus [48].

#### 3.3.2. ROS and HIF-1*α*

The presence of a hypoxic environment is often associated with the longer survival of cancer cells. At the level of the tumor microenvironment, hypoxia in turn causes an increased basal metabolism of the cancer cell which, consequently, becomes able to self-feed the same hypoxia, especially through activation of the hypoxia inducible factor 1 *α* (HIF-1*α*). [49]. ROS signaling via HIF-1*α* is a crucial step in promoting cellular proliferation and tumorigenesis; growing evidence suggests that a number of intermediates, such as PI3K/Akt or ERK, play important roles in ROS mediated HIF-1*α* regulation [50], while also promoting the EMT. Therefore, targeting specific mediators in these regulatory pathways may serve as novel therapeutic approaches for cancer treatment [51].

## 4. TGF-*β* and Oxidative Stress Crosstalk in Cancer Cell Metabolism

It has been widely demonstrated that TGF-*β* is able to regulate ROS levels; on the one hand by increasing its production, on the other hand by inhibiting the activity of antioxidant or scavenging systems at a cellular level [52,53]. Moreover, the increased level of ROS can in turn induce the expression of TGF-*β* and stimulate its release, thus making it active [52,54]. At the same time, it is known that ROS are mediators of many effects exerted by TGF-*β*, which in turn can modulate ROS levels by increasing their production and reducing the activity of antioxidant systems.

**Figure 1:**
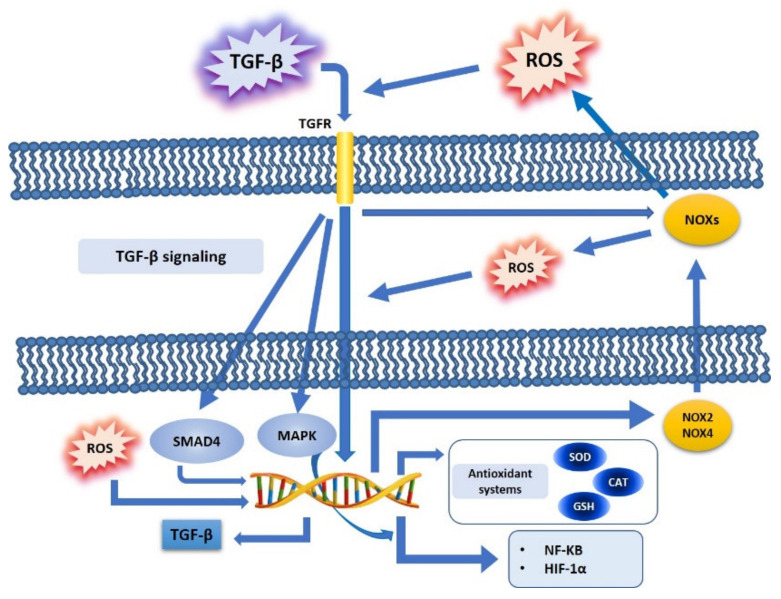
TGF-*β* and ROS crosstalk. TGF-*β* binds its cell surface receptors and induces the activation of downstream pathways (SMAD, MAPK), and further regulates ROS production (NOXs activation, NOX 2 and 4)) or modulates antioxidant systems (SOD, CAT, GSH) and redox-sensitive transcription factors (NF-kB, HIF-1*α*). Furthermore, increased ROS production may directly induce TGF-*β* expression at a nuclear level.

In tumor cells, it has been demonstrated that there is a close correlation between oxidative stress and TGF-*β* (Figure 1). ROS has been shown to mediate many effects of TGF-*β* in cancer progression, by deregulating tumor suppressors and, at the same time, by promoting tumorigenesis; one of the mechanisms of crosstalk between ROS and TGF-*β* in cancer cell metabolism shows ROS as directly involved in the regulation of the TGF-*β* pathway, thus involving factors such as SMAD, MAPK and NF-kB, and in turn promoters of cell proliferation and motility [55], particularly by increasing SMAD and thus making tumor cells resistant to the inhibition of proliferation exerted by TGF-*β* in the initial step of tumorigenesis [56]. Furthermore, in cancer cells TGF-*β* is involved in many other signaling pathways, such as PI3K/Akt or MAPK, which, in turn, regulate redox-sensitive transcription factors, such as NF-kB or HIF-1*α* [57].

### 4.1. TGF-β, ROS, and NOX Family

The NOX family plays an important role in mediating the actions of TGF-*β* via the ROS produced, and this effect is found to be deregulated in tumors. In particular, ROS generated by NOX4 mediate apoptosis, which in turn is mediated by the TGF-*β* signaling pathway [58]: it has been shown that NOX4 can mediate TGF-*β* effects, while NOX-dependent redox signaling can regulate TGF-*β* signaling. TGF-*β* activates NOXs via Rac1 [59]: it has been shown that this factor induces NOX4 in different cell types, both in vivo and in vitro [52]. However, the induction of NOX4 gene expression by TGF-*β* particularly occurs via SMAD3, and this effect has been shown in breast cancer cells [60]. Furthermore, TGF-*β* has been demonstrated to induce NOX4 gene expression in crosstalk with an increase in ROS production, while NOX4 is downregulated with reduced ROS synthesis, thus indicating, in pancreatic cancer cells, that NOX4 is the main source of ROS [61]. Finally, it has been shown, in adenocarcinoma Hela cells, that TGF-*β* can also induce NOX2 gene expression and its activation [62] (Figure 1).

TGF-*β* induces and activates NOXs with different effects, depending on the member of the NOX family. NOX4 induction by TGF-*β* may mediate some of its suppressor effects, such as apoptosis or senescence: it has been shown that TGF-*β* induces senescence in hepatocellular carcinoma cells and inhibits tumor growth [63]. This mechanism has been explained as mediated by the liver-specific tumor suppressor STAT5, which in turn controls NOX4 expression and, consequently, drives the proapoptotic proteins PUMA and BIM in mice [64], as NOX4 upregulation via TGF-*β* in hepatocytes is required for its pro-apoptotic activity [65]. On the contrary, other members of the family, dependent on Rac1 and activated by TGF-*β*, might have opposite effects [59], such as inhibition of the epidermal growth factor (EGF) pathway, which enhances TGF-*β* induced apoptosis in rat hepatoma cells, thus inducing oxidative stress via increased ROS production, coincident with a change in the expression pattern of the NOX isoforms [66,67].

### 4.2. TGF-β, ROS, and Antioxidant Systems

TGF-*β* is able to increase ROS levels by downregulating the expression of antioxidant systems. TGF-*β* regulates the activity of ROS, both by modulating their production and by downregulating the expression of antioxidant enzymes, such as SOD and CAT [52,68], and by modulating cellular antioxidant molecules, such as GSH [52].

Concerning SOD and CAT, it has been shown that these antioxidant enzymes are downregulated in many tumors [32,36], and some papers have demonstrated that TGF-*β* is the mediator in suppressing the expression of Mn-SOD and CAT [68].

It was shown that TGF-*β* induced a decrease in the concentration of GSH, inhibiting the expression of the catalytic subunit of the gamma-glutamylcysteine synthetase (GLC) enzyme: TGF-*β*, by inhibiting GLC expression, causes a strong reduction of GLC activity, thus also promoting GSH inhibition in the human alveolar epithelial cells of lung adenocarcinoma [69] (Figure 1).

### 4.3. TGF-β, ROS, and Redox-Sensitive Factors

TGF-β is linked to NF-kB in a ROS dependent way [59]: TGF-*β* activates NF-kB in a ROS dependent manner, and in turn ROS modulate the NF-kB signaling pathway, especially by increasing the IKK/NEMO dimerization, an event mediated by ROS-sensitive IKK phosphatases. In this way, active IKK phosphorylates IKB, thus inducing nuclear translocation and activation of NF-kB [47] (Figure 1).

Concerning HIF-1*α*, it has been shown that this factor induces TGF-*β* production, and that, at the same time, hypoxia stabilizes HIF-1*α*, mainly by promoting EMT and related cancer metastasis [70]: ROS are not only produced by an aberrant function of mitochondrial complex III during hypoxic stress, but ROS are also stabilized by HIF-1*α,* thus interacting with Snail during EMT [55], in order to enhance the invasiveness in cancer cells [71] (Figure 1).

### 4.4. TGF-β, ROS, and the Epithelial Mesenchymal Transition (EMT)

The EMT is an event characterized on the one hand by the loss of polarity and cell junctions of the epithelial cells, and on the other hand by an increased cellular motility and the acquisition of a mesenchymal phenotype, which gives rise to embryonic development and tissue reconstruction under normal physiological conditions. However, this process can be pathological, as EMT drives fibrosis development and cancer. It is commonly accepted that TGF-*β*-induced EMT is a key step in mediating tumor invasiveness and metastasis [72]. 

TGF-*β* induces the EMT of transformed cells, and this event makes the tumor capable of invading surrounding tissues and promoting metastases. In fact, EMT represents one of the key steps that drive the acquisition of a metastatic phenotype in cancer cells, strongly collaborating in the pathogenesis of cancer and the tumor microenvironment [73]. The ability of TGF-*β* to induce EMT is a crosstalk with ROS: ROS synergize with TGF-*β* both in the initiation and progression of cancer via EMT, not only by increasing the redox imbalance in tumor cells, but also by co-inducing EMT, which is then hyperactivated in carcinoma cells [74].

Therefore, it has been shown that TGF-*β* and ROS participate in inducing EMT: several studies have demonstrated that, in tumor cells incubated with antioxidant treatments, the ability of TGF-*β* to induce EMT is decreased, suggesting the crucial role exerted by oxidative stress. Furthermore, ROS production, whether TGF-*β*-dependent or not, can increase TGF-*β* expression, which in turn may contribute to the development of EMT [75].

While the link between carcinogenesis and oxidative stress, through chronic inflammation, has found support in research studies on a wide range of tissue types, the link between ROS and EMT is less well defined. ROS produced at a mitochondrial level, via MMP-3/Rac1b, can induce EMT with loss of E-cadherin and an increase of vimentin and Snail, and with a consequent increase in invasiveness and motility [76]. Cannito et al. [77] demonstrated that ROS are crucial in the early stages of EMT, in the crosstalk with hypoxia, in hepatoblastoma, colon, and pancreas cell lines [77]: it has been shown that the EMT induced by hypoxia is related to an increased intracellular ROS production, which in turn leads to GSK-3*β* phosphorylation and inactivation, and in turn is linked to the SMAD-mediated TGF-*β* pathway. Recently, it has been demonstrated in human epithelial cells that low doses of H_2_O_2_ induce EMT by downregulation of E-cadherin and Zonula Occludens 1 expression, with a simultaneous upregulation of α-SMA, and by promoting EMT [78].

**Figure 2:**
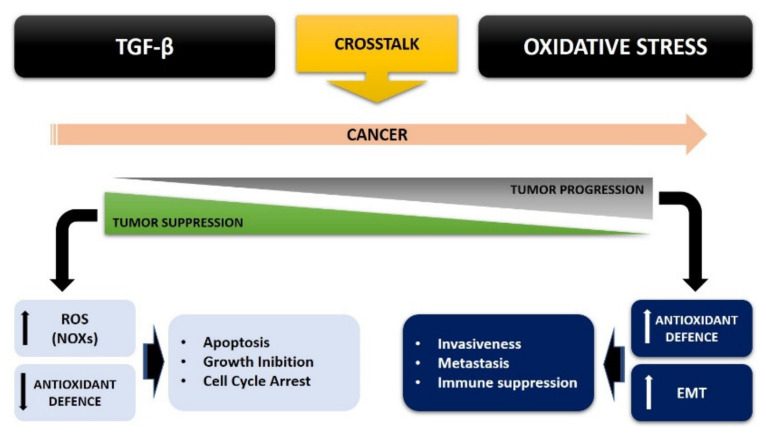
TGF-*β* and oxidative stress crosstalk in cancer cells.

The control of cellular redox homeostasis appears to be related to EMT, and oxidative stress is an important factor that increases tumor malignancy [79]. Oxidative stress can in turn modify the cellular response to TGF-*β*, in a growing crosstalk that increasingly leads cancer cells to be aggressive and more invasive. This TGF-*β* overexpression may in turn be regulated by ROS production within the cancer, via both a SMAD and non-SMAD signaling pathway, leading to EMT (Figure 2).

### 4.5. TGF-β and ROS Crosstalk in Cancer: A Possible Therapeutic Approach

To date, several studies have been undertaken seeking a possible therapeutic approach to cancer by exploiting the studies done on oxidative stress and the TGF-*β* pathway. In recent years, some antioxidants (vitamins C and E) have been proposed as potential drugs able to counteract oxidative stress in cancer development [80]. However, most antioxidants taken orally have limited absorption profile, which leads to low bioavailability and insufficient concentrations at the target site [80,81]. Therefore, some studies have performed experiments with specific nanoparticles with intrinsic antioxidant properties and also functionalized for targeted therapy. Finally, the use of antioxidants, such as N-acetylcysteine or NOX inhibitors, in cancer cells has been demonstrated to be efficient in inhibiting cell proliferation, invasion, and metastasis [80,81].

Similarly, other studies have identified the TGF-*β* pathway as a target for an anti-cancer pharmacological approach, particularly concerning the role of TGF-*β* in mediating the immunosuppression of immune cells and promoting angiogenesis and EMT in cancer cells [4]. It has been showed that a T cell specific blockade of TGF-*β* signaling could enhance anti-tumor immunity via a cytotoxic T cell response [82], while recently, some studies have demonstrated that the overexpression of E-cadherin, the main epithelial marker of EMT downregulated in cancer cells, could suppress cellular migration and invasiveness, while the inhibition of N-cadherin, a mesenchymal marker of EMT overexpressed in cancer cells, caused the opposite effects [83].

However, it would be useful to combine these different therapeutic approaches in cancer treatment, in an attempt, given the clear crosstalk between oxidative stress and TGF-*β*, to improve cancer treatment.

## 5. Conclusions

Therefore, the crosstalk between ROS and TGF-*β* strongly contributes to tumorigenesis, thus bypassing the inhibition of cell proliferation and thus increasing the malignancy of tumor cells. The mutual interaction between oxidative stress and TGF-*β* plays a role in almost all stages of tumor development: this interplay can therefore affect the initial antitumoral role exerted by TGF-*β* in the initial phase of tumor progression, thus becoming pro-tumorigenic and promoting the malignant phenotype of cancer (Figure 2).

Therefore, a crosstalk between oxidative stress/ROS and TGF-*β* exists in cancer cells: TGF-*β* regulates oxidative stress by increasing ROS production and regulating the antioxidant systems, and at the same time ROS regulate the TGF-*β* signaling pathway, especially promoting EMT, which contributes to cancer invasiveness. Furthermore, ROS and TGF-*β* can cooperate in modulating inflammatory and immune response against cancer, thus creating a microtumor environment that benefits cancer cells, to survive and induce a resistance of the tumor cells to apoptosis and chemotherapy. Both ROS and TGF-*β* have important roles in the response of the innate immune system, allowing cancer cells to escape immune surveillance and increasing tumor growth and development, by inducing EMT together and strongly improving tumor progression. Furthermore, TGF-*β* is involved in multiple signaling pathways regulated by redox homeostasis in cancer, through the regulation of redox-sensitive transcription factors, such as NF-kB and HIF-1*α*.

Therefore, this ROS/TGF-*β* crosstalk strongly contributes to tumorigenesis, by avoiding the inhibition of cell proliferation and increasing the malignancy of tumor cells, where oxidative stress can convert the antitumor role of TGF-*β* in early tumor progression into a pro-tumorigenic role, thus signaling a malignant phenotype of cancer.

Take as a whole, this review tries to better understand this crosstalk between TGF-*β* and oxidative stress in cancer cell metabolism, in an attempt to improve the pharmacological and therapeutic approach against cancer, and also considering the growing interest in clarifying this important topic.

## Data Availability

Not applicable.
